# The spectrum effect in tests for risk prediction, screening, and diagnosis

**DOI:** 10.1136/bmj.i3139

**Published:** 2016-06-22

**Authors:** Juliet A Usher-Smith, Stephen J Sharp,, Simon J Griffin

**Affiliations:** 1 The Primary Care Unit, University of Cambridge, Strangeways Research Laboratory, Cambridge, CB1 8RN, UK; 2 MRC Epidemiology Unit, University of Cambridge, Institute of Metabolic Science, Cambridge, UK

## Abstract

The spectrum effect describes the variation between settings in performance of tests used to predict, screen for, and diagnose disease. In particular, the predictive use of a test may be different when it is applied in a general population rather than in the study sample in which it was first developed. This article discusses the impact of the spectrum effect on measures of test performance, and its implications for the development, evaluation, application, and implementation of such tests.

**Summary points**

The spectrum effect describes the variation in performance of tests for prediction, screening, and diagnosis of disease among different population subgroups
A test developed in a population with a higher prevalence of disease (or at higher risk) will typically have a lower sensitivity and higher specificity when applied in a population with lower disease prevalence (or at lower risk)
Care should be taken when comparing the performance of tests developed and evaluated in different populations and using different methods
Ideally new tests should be developed and evaluated using data from the population(s) in which they are intended to be used

Much of clinical practice relies on using tests that measure one or more characteristics of an individual to determine whether that individual is at risk of developing a condition of interest, or does or does not have a particular disease. These tests include risk prediction scores, screening tests, and diagnostic investigations. When evaluating the clinical performance of such tests, the most commonly used measures are the sensitivity, specificity, predictive value, likelihood ratio, and area under the receiver operating characteristic curve (box 1). The likelihood ratio is multiplied by the pre-test odds of the condition to obtain the post-test odds, which can be converted to a post-test probability (known as the positive predictive value). The positive predictive value is well known to vary with the prevalence of the condition. However, while the variation in likelihood ratio, sensitivity, and specificity due to the phenomenon known as the “spectrum effect” or “spectrum bias” has been well described,^1^ ^2^ ^3^ ^4^ ^5^ it is frequently overlooked by the wider medical and policy-making community when interpreting data on test performance.

**Box 1: Commonly used measures for evaluating the clinical performance of diagnostic and screening tests**

- Sensitivity=the probability that an individual with the disease has a positive test result
- Specificity=the probability that an individual without the disease has a negative test result
- Positive predictive value=the probability that an individual with a positive test result has the disease
- Negative predictive value=the probability that an individual with a negative test result does not have the disease
- Positive likelihood ratio=the probability that an individual with the disease has a positive test result, divided by the probability that an individual without the disease has a positive test result (that is, sensitivity/(1−specificity))
- Negative likelihood ratio=the probability that an individual with the disease has a negative test result, divided by the probability that an individual without the disease has a negative test result (that is, (1−sensitivity)/specificity)
- Area under the receiver operating characteristic curve (AUROC)=the probability that a classifier will correctly rank a randomly chosen person with the disease higher than a randomly chosen person without the disease (that is, the area under a plot of sensitivity against (1−specificity))

In this article, we aim to highlight the importance of this effect to all those developing, evaluating, or implementing tests for prediction, screening, or diagnosis. We begin by discussing the spectrum effect in general with examples taken from the literature, and then use simulations to illustrate variation in the likelihood ratio, sensitivity, and specificity with prevalence and population distribution of the condition. Finally, we discuss the importance of considering the spectrum effect in the development, evaluation, and choice of tests.

## What is the spectrum effect and why does it arise?

When Ransohoff and Feinstein first described the spectrum effect in 1978,^5^ they observed that the performance of a test in practice might be misrepresented by clinical studies that include too narrow a range of individuals with or without the disease of interest. Although they did not use the term “spectrum bias” explicitly, their discussion of a patient spectrum in a paper examining biases affecting diagnostic test research led to the use of the term to describe subgroup variation. This term “spectrum bias” is still used in the literature. We and others,^6^ however, prefer the term “spectrum effect,” because it better reflects the inherent nature of variation in test performance among population subgroups.

The implication of the spectrum effect is that the most commonly used measures of the performance of tests (sensitivity, specificity, and likelihood ratios) vary with the prevalence and distribution of disease in a population or sample. Only in situations with a truly dichotomous disease status or perfect test and an equal probability of diagnostic misclassification in individuals with and without the disease are these measures independent. These conditions are rarely met. Many situations have a continuum of (measurable or unmeasurable) traits on which the classification of disease status is based, varying from clear absence to clear presence of the disease.^7^

Further, all tests are subject to error, including:

- Simple measurement error (eg, measurement of plasma glucose in the screening for type 2 diabetes, or measurement of D-dimer to guide further investigations for suspected deep vein thrombosis or pulmonary embolus)
- Subjective error when categorisations are based on patient recall, clinical symptoms, examination findings, radiological images, or histological specimens that healthcare professionals could interpret differently (eg, colonoscopic identification of advanced adenoma or mammography for breast cancer)
- The influence of unmeasured covariates on test results.

This last source of error arises because few markers are specific to one disease, and so false positives are generated by conditions very close to but different from the target condition. For example, B type natriuretic peptide can contribute to the diagnosis of heart failure, but might also be raised in several other conditions such as renal failure and pulmonary hypertension. 

The consequence of this combination of error in the test result and the continuum of true disease status is that the probability of misclassification of disease status varies between individuals with individuals with true values of the underlying characteristics close to the cut-off point for diagnosis more likely to be misclassified than others.

## How does the spectrum effect influence tests?

The effect of these errors on overall test performance depends on the proportion of individuals misclassified, which in turn depends on the number of individuals close to the cut-off point of the test. Therefore, the performance of tests is influenced by both the prevalence of the condition or disease in the sample in which it is assessed and the characteristics of the sample. Using an approach similar to that demonstrated by Brenner and Gefeller,^8^ these effects can be illustrated by a simulated situation in which there is a continuous variable X with a value for each individual, and true disease status is defined by the value of X being above or below a particular threshold value (supplementary appendix).

X could represent an underlying trait—for example, true fasting glucose, for which the disease is diabetes and the threshold value for X is 7.0 mmol/L, or true systolic blood pressure, for which the disease is hypertension and the threshold value is 150 mm Hg. However, X could also represent true underlying risk of a disease—for example, true underlying 10 year risk of cardiovascular disease can be classified as “high risk” or “low risk” on the basis of a threshold value for X. This particular threshold is currently 10% in England and Wales, as recommended by the National Institute for Health and Care Excellence to decide whether to offer statins.^9^

In all these examples, the true value of X for an individual is unknown, but it is possible to obtain an estimate (Z) of X, either by measurement of the trait of interest (fasting plasma glucose, systolic blood pressure), or by calculation from a risk prediction model (10 year cardiovascular risk). Z is unlikely to be exactly equal to X for all individuals, because of measurement error if X represents a measurable trait, or prediction error if X represents an underlying risk.

By varying the assumptions about the distribution of X, it is possible to explore the variation in the performance of a test based on Z in populations with different underlying prevalence of disease or distributions of risk.

### Scenario 1

The prevalence of disease in the population can be varied by changing the mean of X while keeping the threshold value constant (fig 1). As prevalence decreases, the positive likelihood ratio (LR+), negative likelihood ratio (LR−), and specificity increase, while sensitivity decreases, with 10-fold changes in likelihood ratios and variation of 30% in sensitivity and specificity (fig 1A and 1B[Fig f1]). Change in prevalence is equivalent to performing a test in different health settings. A diagnostic procedure developed in secondary care among patients suspected of having a particular disease will typically have lower sensitivity and higher specificity when applied as a screening tool in primary care where disease prevalence is lower. The same effect is seen when applying the test in subgroups known to be at higher risk (the referral filter^3^). For example, if a new test for diabetes is evaluated in routine clinical practice in primary care, it is likely that general practitioners would initially test people with symptoms or risk factors. If the test had been designed to be used in such a high risk group then that approach would be appropriate, but if the test had been designed for population based screening of asymptomatic individuals, the specificity in that high risk population will be lower—and the sensitivity higher—than estimated in the original development population.

**Figure f1:**
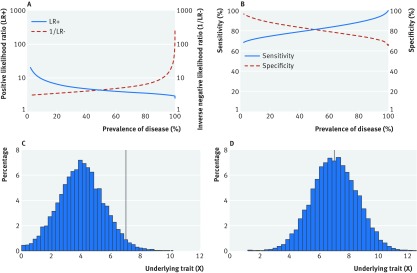
**Fig 1** Scenario 1. Variation in (A) positive likelihood ratio (blue solid line) and inverse of negative likelihood ratio (red dashed line), and (B) sensitivity (blue solid line) and specificity (red dashed line) with true prevalence of a disease where true prevalence of disease is changed by varying the mean of a normal distribution of a continuous variable X while keeping the threshold value constant (C and D). True disease is defined as present if X≥7, and absent if X<7. Disease prevalence, for illustration, is (C) 2.3%, (D) 49.6%. As prevalence decreases, the positive likelihood ratio (LR+), negative likelihood ratio (LR−), and specificity increase, while sensitivity decreases, with 10-fold changes in likelihood ratios and variation of 30% in sensitivity and specificity. Values for all plots obtained via simulation as described in the supplementary appendix (scenario 1)

### Scenario 2

The prevalence of disease within the population can also be changed by altering the distribution of X. We did this by simulating a bimodal distribution for X illustrating different potential situations from one in which only a small proportion of the overall population has the disease, to one in which nearly half the population has the disease (fig 2). When X has a distribution as shown in figure 2C[Fig f2], a greater proportion of people with the disease will have a value of X close to the threshold (and hence be at risk of test misclassification) than the corresponding proportion in a distribution as shown in figure 2D[Fig f2]. In other words, as the proportion of the overall population with the disease increases, the risk of test misclassification among those with the disease decreases and—with this particular underlying distribution of X—the result is an increase in sensitivity and decrease in the negative likelihood ratio (fig 2A and 2B[Fig f2]). The wide dispersion of the values of X in this bimodal distribution also means that in figure 2C and 2D[Fig f2], there are fewer individuals with values close to the threshold than in scenario 1 (fig 1C and 1D[Fig f1]). The result is a higher sensitivity and specificity for the same prevalence. For example, when 20% of individuals have the disease, the sensitivity and specificity in scenario 1 are 85% and 95%, respectively, compared with the corresponding values of 98% and 99% in scenario 2. Situations like those shown in figure 2 could arise in a study in which individuals with and without disease are selected from different populations, for example, a case-control study with cases identified from hospital and controls from the community.

**Figure f2:**
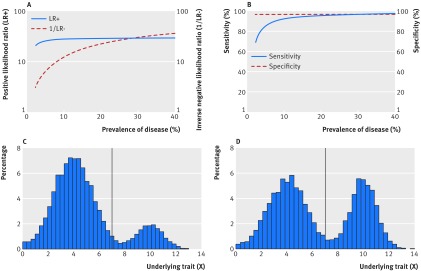
**Fig 2** Scenario 2. Variation in (A) positive likelihood ratio (blue solid line) and inverse of negative likelihood ratio (red dashed line), and (B) sensitivity (blue solid line) and specificity (red dashed line) with true prevalence of a disease where true prevalence of disease is changed by altering the distribution of a bimodal distribution of a continuous variable X while keeping the threshold value constant (C and D). True disease is defined as present if X≥7, and absent if X<7. Disease prevalence, for illustration, is: (C) 17.1%, (D) 41.1%. As prevalence increases, the risk of test misclassification among people with the disease decreases and—with this particular underlying distribution of X—results in an increase in sensitivity and decrease in the negative likelihood ratio. Values for all plots obtained via simulation as described in the supplementary appendix (scenario 2)

This example also illustrates how the sensitivity is affected by the stage and severity of disease or condition in the population (patient spectrum). The more advanced or severe the disease or condition is among those individuals affected, the greater the separation in the measured variable(s) between people those with and without the condition, and the better the test will appear to be at discriminating between the two groups. For example, a hypothetical pregnancy test would appear to perform better if evaluated among a cohort of individuals attending an antenatal clinic than if evaluated in the wider community. The antenatal clinic cohort would include women who most likely would either be definitely pregnant or very likely not to be pregnant if they were friends or relatives of the pregnant women, whereas the wider community may include women in the very early stages of pregnancy. 

## Importance of the spectrum effect in practice

Although the examples given above are for a situation in which the target condition is based on measurement of a continuous variable, with the presence or absence of the condition based on the value of that variable being above or below a threshold, apparent differences in test performance arising from variation in prevalence and study populations are widespread and can be large enough to influence clinical practice. In a review of 23 meta-analyses of diagnostic test accuracy, statistically significant associations between prevalence and sensitivity or specificity were found in one of three reviews, with specificity decreasing by between 0.1% and 0.5% for every 1 percentage point increase in prevalence in pooled analysis.^10^ Many examples also illustrate the variation with the affected population’s characteristics. For example, Moons and colleagues^4^ demonstrated that the performance of an exercise test for coronary artery disease varied across patients with different characteristics; the positive likelihood ratio was 3.8 in individuals with systolic blood pressure 141-240 mm Hg, and 17.0 in those with systolic blood pressure 100-140 mm Hg.

## Implications for research, clinical practice, and policy

As for prognostic models,^11^ ^12^ ^13^ ^14^ the development and evaluation of screening and diagnostic tests should ideally be undertaken by use of data from two different cohort studies (one for development and one for evaluation), which include a wide range of disease states and population characteristics. This approach would allow for some exploration of the spectrum effect, and ensure that the test’s performance is not overestimated by use of a sample that is unrepresentative of the wider population. If this is not possible and case-control studies are used, ideally the study sample should be as similar as possible to the population in which the test is intended to be used.

In all situations, however, researchers should report sufficient data to allow the extent of the potential spectrum effect to be assessed. The STARD statement for reporting studies of diagnostic accuracy^15^ recommends that authors describe the study participants in detail, including the distribution of disease severity. Authors should also report estimates of variability of diagnostic accuracy between readers, centres, or subgroups of participants and estimates of test reproducibility, to allow readers to assess any potential spectrum effect. Subgroup variation in sensitivity and specificity estimates can be examined by stratification of the characteristic defining the subgroup and by a simple χ^2^ test of association. Subgroup specific receiver operating characteristic (ROC) curves can also be constructed and compared, while calculating the critical ratio (the difference in area under the ROC curves divided by the standard deviation of the difference) and comparing it with a normal distribution can provide a quantitative comparison of these ROC curves.^16^ As Mulherin and Miller point out,^6^ logistic modelling can produce estimates of test performance^17^ among small groups, and facilitates investigation of patient characteristics that are multicategorical or continuous, enabling the investigator to model separately factors that affect sensitivity and specificity.^4^

Care should also be taken by researchers and policy makers when comparing the performance of diagnostic tests developed in different populations using different methods. For example, it is not appropriate to compare the sensitivity and specificity of a new diagnostic test developed and validated in a secondary care based case-control study with the sensitivity and specificity of screening programmes in a national population.

## Conclusion and implications

When reviewing a study of a new risk prediction, screening, or diagnostic test and deciding whether to use that test in practice, clinicians and policymakers should examine the relevance of the study sample to their own population. Ideally new tests should be developed and evaluated using data from the population(s) in which they are intended to be used. If not possible, it is important to remember that, as a result of the spectrum effect, calculations of positive and negative predictive value will only partly adjust for differences in disease prevalence (box 2). The performance of the test in their own population might, therefore, be different from that reported in the study, and the potential health gains associated with introduction of the test should be interpreted with caution.

**Box 2: Use of the spectrum effect when considering new diagnostic tests in clinical practice: hypothetical example**

Dr B, a primary care physician, is considering introducing into his practice a new test for bacterial infections in patients with upper respiratory tract symptoms. Dr B finds two published reports which have evaluated the test’s performance. In the first report, the test was evaluated in a case-control study with 40 patients with bacterial infection identified from a hospital ear nose and throat clinic and 60 controls attending the orthopaedic clinic in the same hospital. The sensitivity and specificity were reported to be 97% and 97%, respectively (fig 2B[Fig f2]). Assuming that the prevalence of bacterial infection in patients with upper respiratory tract symptoms in primary care is about 10%, if Dr B performs the test on a patient and the result is positive, there is a 78% probability that the patient has a bacterial infection and that antibiotics might be of benefit.

In the second report, the same test was evaluated in a cross-sectional study in the general population in which the prevalence of bacterial infections was 10%. The sensitivity and specificity were reported to be 72% and 92%, respectively (fig 1B[Fig f1]). Using these values instead, if Dr B performs the test on a patient and the result is positive, there is only a 50% probability that the patient has a bacterial infection.

## Web Extra

Extra material supplied by the author

**Supplementary appendix:** Spectrum effect

## Notes

We thank Gary Abel for helpful discussions and *The BMJ* reviewers for their helpful comments and suggestions.

Provenance and peer review: Not commissioned; externally peer reviewed.
