# Supplementary material for: The spectrum effect in tests for risk prediction, screening, and diagnosis
Source: BMJ. 2016 Jun 22;353:i3139. doi: 10.1136/bmj.i3139 (PMC4916916; doi:10.1136/bmj.i3139)
Supplement: Supplementary file 1 — Supplementary appendix: Spectrum effect [file ushj028979.ww_default.pdf]

Supplementary appendix: Spectrum effect

X is a continuous variable representing an underlying trait, such as fasting plasma glucose or systolic blood pressure.

c is a fixed “threshold” value whereby if $X \geq c$, a particular disease is present, and if $X < c$, that disease is absent.

X represents the “true” value of the trait, which would usually be unknown for an individual. There is random error in the measurement of X for an individual. This random error (denoted e) is assumed to have a normal distribution, with mean 0 and standard deviation σ_E , and is assumed to be independent of X.

Therefore if Z is a continuous variable representing the measured values of the trait for each individual, $Z = X + e$.

Scenario 1.

X is assumed to have a normal distribution with mean m and standard deviation σ_X. The following (arbitrary) values were chosen for the various parameters:

$\sigma_E = 0.5$
$c = 7$
$\sigma_X = 1.5$
m = 4 + i, where i=0, 0.05, 0.1, 0.15, ... , 8.

For each value of i, 1000 datasets were created, each containing the values of X and Z for 10000 individuals. Simulated distributions were truncated at 0, so it was not possible for X or Z to have a negative value. For each dataset the sensitivity, specificity, LR+ and (1/LR-) values were calculated for a diagnostic test based on Z and c. Median values of each of these quantities over the 1000 simulated datasets were calculated, and plotted against the true prevalence of disease in the population.

Scenario 2.

X is assumed to have a bimodal distribution. In (10000-j) individuals, values from a normal distribution with mean m=4 and standard deviation $\sigma_X = 1.5$ were simulated, while in j individuals, values from a normal distribution with mean m=10 and standard deviation $\sigma_X = 1$ were simulated. Values of j from 0 to 4000 were used. Values assumed for σ_E and c, number of datasets simulated, and subsequent calculations of sensitivity, specificity, LR+ and (1/LR-), were as in Scenario 1.
